# Collagen transport and related pathways in Osteogenesis Imperfecta

**DOI:** 10.1007/s00439-021-02302-2

**Published:** 2021-06-24

**Authors:** Lauria Claeys, Silvia Storoni, Marelise Eekhoff, Mariet Elting, Lisanne Wisse, Gerard Pals, Nathalie Bravenboer, Alessandra Maugeri, Dimitra Micha

**Affiliations:** 1 Department of Clinical Genetics, Amsterdam UMC, Amsterdam Movement Sciences, Vrije Universiteit Amsterdam, Amsterdam, The Netherlands; 2 Department of Internal Medicine Section Endocrinology, Amsterdam UMC, Amsterdam Movement Sciences, Vrije Universiteit Amsterdam, Amsterdam, The Netherlands; 3 Department of Clinical Chemistry, Amsterdam /UMC, Amsterdam Movement Sciences, Vrije Universiteit Amsterdam, Amsterdam, The Netherlands

## Abstract

Osteogenesis Imperfecta (OI) comprises a heterogeneous group of patients who share bone fragility and deformities as the main characteristics, albeit with different degrees of severity. Phenotypic variation also exists in other connective tissue aspects of the disease, complicating disease classification and disease course prediction. Although collagen type I defects are long established as the primary cause of the bone pathology, we are still far from comprehending the complete mechanism. In the last years, the advent of next generation sequencing has triggered the discovery of many new genetic causes for OI, helping to draw its molecular landscape. It has become clear that, in addition to collagen type I genes, OI can be caused by multiple proteins connected to different parts of collagen biosynthesis. The production of collagen entails a complex process, starting from the production of the collagen Iα1 and collagen Iα2 chains in the endoplasmic reticulum, during and after which procollagen is subjected to a plethora of posttranslational modifications by chaperones. After reaching the Golgi organelle, procollagen is destined to the extracellular matrix where it forms collagen fibrils. Recently discovered mutations in components of the retrograde transport of chaperones highlight its emerging role as critical contributor of OI development. This review offers an overview of collagen regulation in the context of recent gene discoveries, emphasizing the significance of transport disruptions in the OI mechanism. We aim to motivate exploration of skeletal fragility in OI from the perspective of these pathways to identify regulatory points which can hint to therapeutic targets.

## Osteogenesis Imperfecta: are we ready to classify it?

Osteogenesis Imperfecta, or brittle bone syndrome, is a rare genetic connective tissue disease. It is characterized by altered microarchitecture of the bone, which leads to fragility (Glorieux 2001). In the more severe cases, patients also manifest a variable degree of skeletal dysplasia and short stature. Patients may also be affected by extraskeletal features such as dentinogenesis imperfecta, hearing loss and blue sclera (Van Dijk et al. 2010). Cardiac valvulopathies and aortic dilatation are one of the most common cardiovascular aspects of OI (Ashournia et al. 2015); altered diastolic function is also reported which may also predispose OI patients to higher risk for cardiovascular disease (Migliaccio et al. 2009; Radunovic and Steine 2015). The prevalence of OI is approximately 1 in 15–20,000, qualifying as one of the most prevalent inherited skeletal disorders. Defects in collagen type I are central in the systemic pathology of the disease.

OI is a phenotypically and molecularly heterogeneous group of disorders (Forlino and Marini 2016). Because of this variability, many researchers have attempted to develop models for its classification in an effort to aid its efficient communication and understanding. The most commonly used classification is the “Sillence classification”, established by David Sillence (Sillence et al. 1979; Van Dijk et al. 2010). This classification initially divided the disease in four different types based on the clinical findings and the mode of inheritance. OI type I is phenotypically classified as the mildest type of OI; this patient group experiences a variable number of bone fractures and usually blue sclerae (Van Dijk and Sillence 2014). This is in great contrast to the most severe phenotype, classified as OI type II, characterized by prenatal onset of multiple fractures and deformity of the long bones and the ribs, resulting in perinatal lethality. In addition, OI type II is subdivided in three classes (A, B and C) based on differences in radiographic features (Gajko-Galicka 2002). Type III is the most severe adult form of OI, with patients suffering from severe gradual deformity of the long bones and/or spine (Gajko-Galicka 2002). The patient group diagnosed with type IV OI is the most phenotypically diverse, from mild to severe. Similar to the other types, the patients have fragile bones with and without progressive deformities of the long bones and spine (Sillence et al. 1979).

## Gene discovery in OI classification

To facilitate the discovery of many new genes associated with OI in the last two decades, this classification has been expanded to approximately 20 different types (Bacon and Crowley 2018; Etich et al. 2020b; Hayat et al. 2020). This genetic classification is based on the causative gene, rather than the clinical presentation. However, the ongoing emerging of new genetic causes made clear that OI is a more complex disorder than originally thought, of which the genotype–phenotype correlation is incompletely understood. Genetic causes of OI present a lot of clinical overlap (Table 1) so the genetic classification does not facilitate efficient communication of the disease, and it does not keep up with gene discovery. For this reason, in 2010 experts proposed to simplify the classification by returning to the four clinical types described by Sillence, with the addition of the clinically distinct type V, which includes moderately affected patients and which is uniquely characterized by hyperplastic callus formation and interosseous membrane ossification; as opposed to the genetic variability of the other types, type V is only caused by one specific mutation in the gene interferon induced transmembrane protein 5, *IFITM5* (Van Dijk et al. 2010). According to this classification, most causative genes lead to moderate–severe phenotypes (Table 1) (Van Dijk and Sillence 2014). It is notable that the five Sillence types are based on overall clinical severity largely depending on skeletal features. There exists incomplete evidence about the correlation between the type of OI and the other systemic features of OI (Lindahl et al. 2015; Zhytnik et al. 2019).

**Table 1:** Sillence Classification expanded to OI type V and atypical OI associated with phenotypes and inheritance pattern of OI causative genes up to date (Van Dijk and Sillence 2014)

Sillence type	Clinical severity	Mutated gene(s)	Protein name	Mode of inh.^a^	Pathway
I	Mild, non deforming	*COL1A1/2* (Sillence et al. 1979)	COL1A1/2	AD	
		*CREB3L1* (Keller et al. 2018)	OASIS	AR	ER stress response
II (A, B and C)	Perinatal lethal	*COL1A1/2* (Sillence et al. 1979)	COL1A1/2	AD	
		*CREB3L1* (Symoens et al. 2013),	OASIS	AR	ER stress response
		*CRTAP* (Morello et al. 2006),	CRTAP		Posttranslational mod
		*KDELR2* (van Dijk et al. 2020),	KDELR2		Retrograde vesicle transp
		*LEPRE1* (Cabral et al. 2007; Moul et al. 2013),	P3H1		Posttranslational mod
		*PPIB* (van Dijk et al. 2009a, b)	CYPB		Posttranslational mod
III	Progressively deforming, severe	*BMP1* (Martínez-Glez et al. 2012),	BMP1	AR	Extracellular processing
		*COL1A1/2* (Sillence et al. 1979)	COL1A1/2	AD	
		*CCDC134* (Dubail et al. 2020),	CCDC134	AR	Regulation of MAPK
		*CREB3L1* (Keller et al. 2018),	OASIS		ER stress response
		*CRTAP* (Baldridge et al. 2008),	CRTAP		Posttranslational mod
		*IFITM5* (Farber et al. 2014),	IFITM5		Matrix mineralization
		*FAM46A* (Doyard et al. 2018),	TENT5A		Unknown
		*FKBP10* (Alanay et al. 2010),	FKBP65		Posttranslational mod
		*KDELR2* (van Dijk et al. 2020),	KDELR2		Retrograde vesicle transp
		*LEPRE1* (Baldridge et al. 2008),	P3H1		Posttranslational mod
		*MBTPS2* (Lindert et al. 2016),	S2P	XL	ER stress response
		*MESD* (Moosa et al. 2019),	MESD	AR	WNT signaling pathway
		*PLOD2* (Zhou et al. 2014),	LH2		Posttranslational mod
		*PPIB* (van Dijk et al. 2009a, b),	CYPB		Posttranslational mod
		*SERPINF1* (Becker et al. 2011),	PEDF		Matrix mineralization
		*SERPINH1* (Christiansen et al. 2010),	HSP47		Posttranslational mod
		*SP7* (Fiscaletti et al. 2018),	OSX		Bone cell diff. and sign
		*TMEM38B* (Volodarsky et al. 2013),	TRIC-B		Posttranslational mod/Ca^2+^ homeostasis
		*WNT1* (Pyott et al. 2013)	WNT1		Bone cell diff. and sign
IV	Moderately to severe deforming, variable	*COL1A1/2* (Sillence et al. 1979)	COL1A1/2	AD	
		*PLS3* (van Dijk et al. 2013)	PLS3	XL	Actin-bundling protein
		*PPIB* (Barnes et al. 2010),	CYPB	AR	Posttranslational mod
		*SP7* (Lapunzina et al. 2010),	OSX		Bone cell diff. and sign
		*SPARC* (Mendoza-Londono et al. 2015),	ON		Extracellular matrix
		*WNT1* (Fahiminiya et al. 2013)	WNT1		Bone cell diff. and sign
V	Moderately deforming with calcification in interosseous membranes	*IFITM5* (Corradi et al. 2015)	IFITM5	AD	Matrix mineralization
Atypical OI		*NBAS* (Balasubramanian et al. 2017)	NBAS	AD	Retrograde transport

^a^Autosomal dominant (AD), autosomal recessive (AR) and recessive X-linked (XL)

Although, based on the current state of knowledge it is still preferable to define OI types clinically, inventorying of the molecular diagnosis complements the clinical evaluation and is also paramount for the expansion of our mechanistic comprehension of the disease. Mutations in several OI-causing genes are divided into two groups, the first group includes mutations leading to haploinsufficiency/quantitative defects in collagen type I, whereas, mutations in the other group lead to structural/qualitative defects (Byers et al. 1991). The state of haploinsufficiency usually arises from indels, splice site and frameshift mutations in the collagen type I genes leading to no or unstable mRNA. Although there are exceptions, a general trend exists for haploinsufficiency leading to the milder type I and IV types. Structural alterations of the type I collagen can be caused by mutations in collagen type I genes (dominant negative effect) or can be the result of the defective modification of collagen chains by mutations in the collagen-processing chaperones such as CRTAP, P3H1 and CYPB (van Dijk et al. 2009a, b). Although the latter mostly correlates with moderate–severe OI (Table 1), the phenotypic effect of structural mutations in collagen genes, such as glycine substitutions, varies depending on their location and nature as well as on the chain type (Raghunath et al. 1994; Xiao et al. 2011).

## Collagen type I is central to the Osteogenesis Imperfecta pathology

The genetic background of OI is heterogeneous and currently consists of around 20 genes which are implicated in the regulation of collagen type I or other aspects of bone biology (Etich et al. 2020a). Approximately 85% of patients with OI possess an autosomal dominant mutation in *COL1A1* or *COL1A2* genes. They encode the α1(I) and α2(I) chains of type I collagen, which is the most abundant protein in bone, skin and tendon extracellular matrices (Forlino and Marini 2016). It can also be found in arteries and the heart ventricles, which accounts for the cardiovascular problems. The remaining 15% of the patients have mutations in genes coding for proteins needed for the transcription [*CREB3L1* (cAMP responsive element binding protein 3-like 1)*, MBTPS2* (membrane-bound transcription factor peptidase site 2)], synthesis [*TMEM38B* (Trimeric intracellular cation channel type B)], posttranslational modification [*LEPRE1* (Leucine Proline-Enriched Proteoglycan (Leprecan) 1), *CRTAP* (Cartilage-Associated Protein)*, PPIB* (Peptidyl–prolyl cis–trans isomerase B)*, PLOD2* (Procollagen–Lysine,2-Oxoglutarate 5-Dioxygenase 2)] and chaperone proteins [*SERPINH1* (Serpin Family H Member 1) and *FKBP10* (FKBP Prolyl Isomerase 10)], retrograde transport [*KDELR2* (KDEL Endoplasmic Reticulum Protein Retention Receptor 2) and *NBAS* (NBAS Subunit Of NRZ Tethering Complex)] and extracellular processing [*BMP1* (Bone Morphogenetic Protein 1)] of procollagen or for bone synthesis, development or structure both with regard to bone cell function and differentiation as well as extracellular matrix (ECM) composition [*SERPINF1* (Serpin Family F Member 1), *IFITM5* (Interferon-Induced Transmembrane Protein 5)*, SPARC* (Secreted Protein Acidic And Cysteine-Rich)*, TENT5A* (Terminal Nucleotidyltransferase 5A)*, PLS3* (Plastin 3)*, WNT1* (Wnt Family Member 1)*, MESD* (Mesoderm Development LRP Chaperone)*, SP7* (Sp7 Transcription Factor), *CCDC134* (Coiled-Coil Domain-Containing 134)]. Mutations in these genes are mostly autosomal recessive; an exception to this are mutations in *IFITM5* (autosomal dominant), *PLS3* and *MBTPS2* (recessive X-linked) (Hanagata 2016). Based on this, OI has been defined as a collagen-related disorder, although the involvement in collagen regulation has not been sufficiently demonstrated for all described genetic causes, for which we rely on future studies (Forlino and Marini 2016). To this end, it is essential to investigate collagen in the bone tissue when abnormalities are not found in fibroblasts, which have been commonly used for these studies, although this is not always feasible. This is owing to the fact that patients are usually reluctant to undergo a bone biopsy unless it is part of a planned surgery (Murakami et al. 2011; van Dijk et al. 2012).

Collagen type I belongs to a large and diverse family of collagens that are present throughout the whole body. Collagens are characterized by a triple helix of (α) polypeptide chains; they can be homotrimeric or heterotrimeric. This means that they are composed of three identical alpha chains (e.g. collagen type III) or alpha chains encoded by different genes (e.g. collagen type I encoded by C*OL1A1* and *COL1A2*) (Mienaltowski and Birk 2014). The amino acid triple helical domain of the collagen type I chain consists of 338 uninterrupted tripeptide units with the sequence of Gly–*X*–*Y*. The *X* and *Y* are often proline and hydroxyproline and exclude cysteine and tryptophan (Byers et al. 1991; Gelse et al. 2003). The triple helix is formed around a central axis, starting from its carboxyl–terminal globular end towards the amino–terminus with a pitch of three amino acids per turn (Byers et al. 1991; Gelse et al. 2003). The glycine residues in every third position, which are positioned centrally in the triple helix, are essential for the correct folding of the triple helix, because glycine is the smallest amino acid and the only one that fits inside the triple helix. Due to this, point mutations, such as glycine substitutions, towards the carboxyl–terminal end of the α1(I) chain lead to more severe phenotypic consequences compared to equivalent mutations closer to the amino–terminus (Byers et al. 1991). This is attributed to the exposure of collagen to excessive posttranslational modification, such as hydroxylation and glycosylation, which takes place as a result of delayed triple helix folding, the extent of which depends on the position of the mutation in relation to the carboxyl–terminus (Byers et al. 1991; Engel and Prockop 1991).

**Fig. 1:**
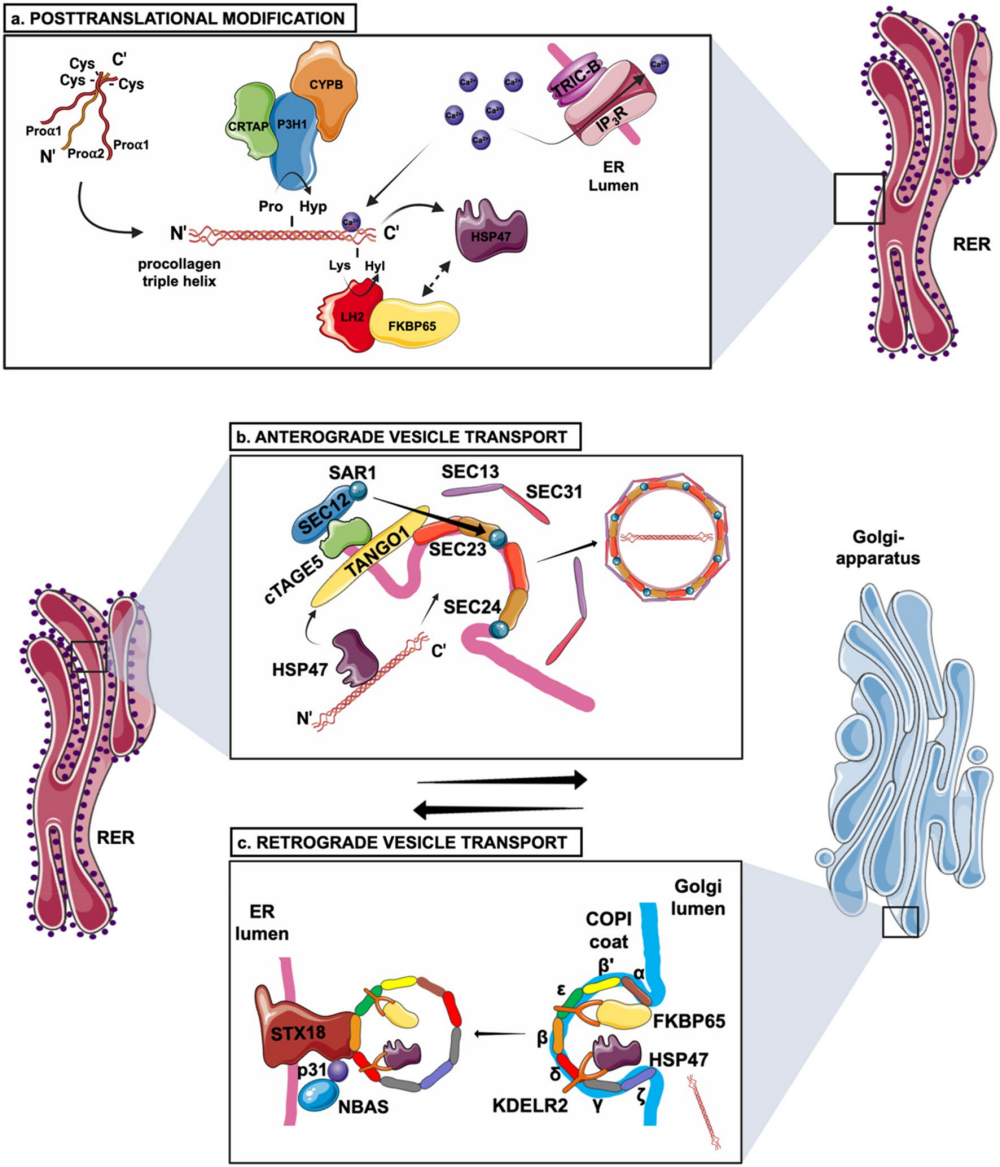

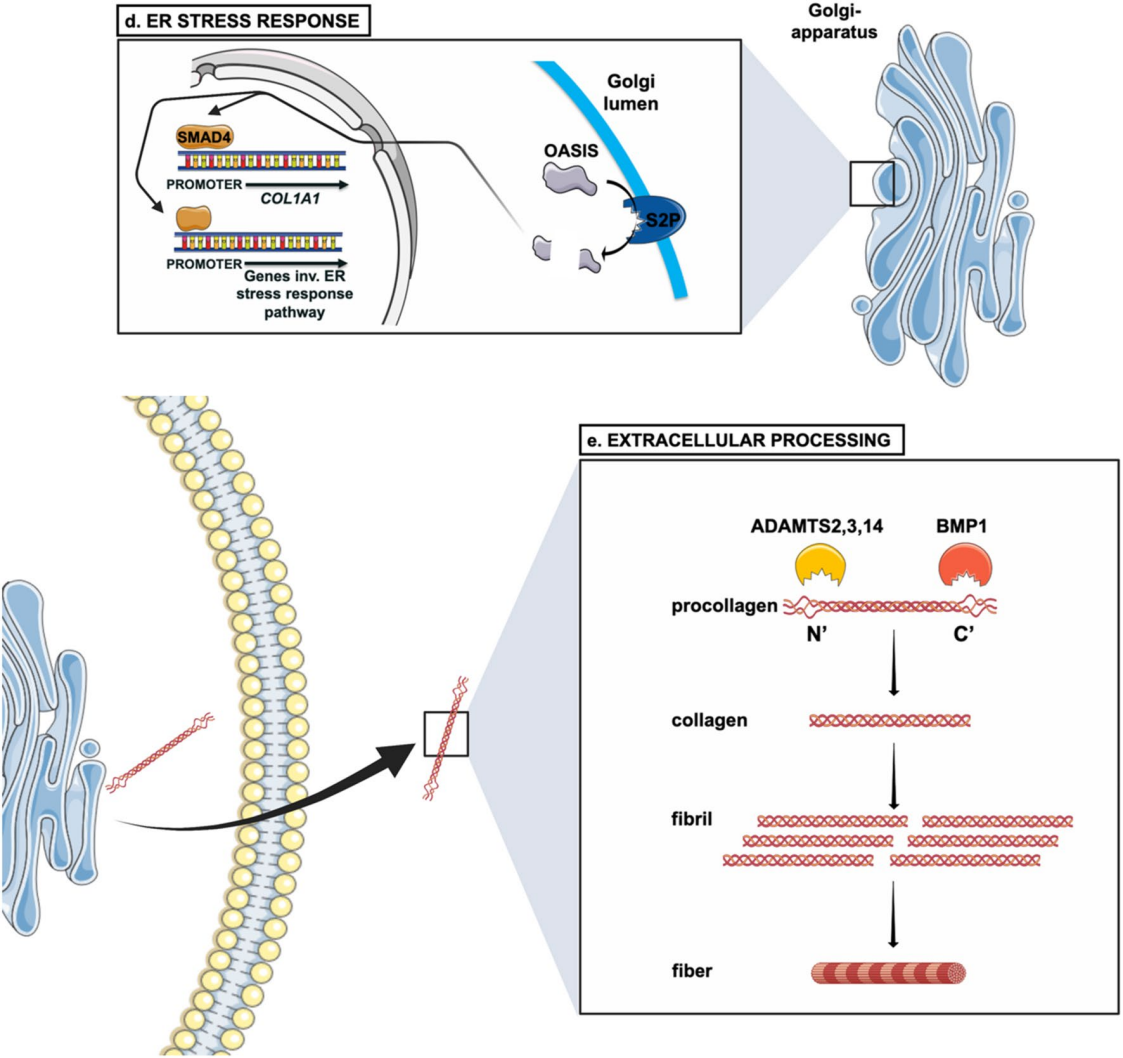
Biosynthesis pathway of collagen type I. **a** Folding and posttranslational modification of the COL1α1 and COL1α2 chains and of the procollagen triple helix takes place in the RER (rough endoplasmic reticulum). These modifications are performed by the CRTAP–P3H3–CYPB and FKBP65–LH2 complexes, turning proline to hydroxyproline and lysine to hydroxylysine respectively. HSP47 is a chaperone protein which assists in the stabilization of the triple helix and its transportation through the ER. The whole process of biosynthesis of type I collagen is calcium-regulated. TRIC-B is one of the channels regulating ER Ca^2+^ flow via IP_3_R channel. Ca^2+^ also stabilizes the process of collagen trimerization and foldings. **b** Anterograde transport of the procollagen triple helix from the ER to the Golgi apparatus via COPII-mediated vesicle transport. Many proteins are involved in the formation of the COPII vesicle such as the SEC23/24 dimer (inner coat), the SEC13/31 dimer (outer coat), SEC12, SAR1, TANGO1 and cTAGE5. TANGO1 and HSP47 facilitate the entering of procollagen in the forming vesicle. SEC12 and in turn SAR1 recruit SEC23/24 dimers to form the inner coat. **c** Retrograde transport of ER proteins via COPI (α-COP, β’-COP, ε-COP, β-COP, δ-COP, γ-COP and ζ-COP) -mediated vesicle transport. FKBP65 and HSP47 are chaperone proteins which are returned to the ER, assisted by KDELR2 receptors in the Golgi membrane. STX18 and NBAS assist in the arrival and fusion of the COPI vesicles to the ER membrane. **d** S2P response to ER stress. The cleavage of OASIS activates transcription (TF: transcription factor) of genes involved in the ER stress response pathway; COL1A1 transcription is activated by SMAD4. **e** Extracellular processing of the procollagen type I triple helix and formation of fibrils and fibers. The C- and N-propeptides are cleaved-off by BMP1 and ADAMTS2, 3 or 14, respectively. After cleavage, the formation of collagen fibrils takes place

Given that collagen type I is the most abundant collagen in the bone tissue, study of its regulation has been the center of OI research. Type I collagen is synthesized as procollagen which is transported from the ER to the Golgi and subsequently to the extracellular space where the mature type I collagen assembles spontaneously into staggered fibril–arrays with diameters between 25 and 400 nm (Gelse et al. 2003; Mienaltowski and Birk 2014). An overview of the collagen biosynthesis, with focus on the proteins connected to OI, can be seen in Fig. 1.

The aim of this review is to shed light on the transport regulation of collagen in OI from the perspective of gene discovery, by summarizing the critical points in this process from which dysregulation can lead to bone fragility. The reader is specifically guided through the modification of collagen in the endoplasmic reticulum (ER) where the contribution of chaperones to this process is explained. The vesicle transport of collagen from the ER to the Golgi is then described by emphasizing how it is facilitated considering the limitations posed by its large molecular size (anterograde transport). An important part of the review are the latest developments on the retrograde transport of chaperones which is appearing as a new mechanism in OI. Next, we cover the current state of knowledge on the extracellular processing of collagen by which it adopts a competent state for fibrillogenesis. The spectrum of skeletal disease presentation is also described as a result of mutations in components of these pathways. Finally, the implications of these findings on collagen transport are related to issues of disease classification and therapeutic prospects.

## ER posttranslational modification

During translation, procollagen type I is placed in the lumen of the ER membrane where many proteins are involved in its posttranslational modification before, during and after triple helix formation which is a key requirement for its correct assembly. Some of those proteins assist for example with the C-terminal propeptide folding and stabilization (BiP, GPR94, Calnexin and PDI) (Lamande and Bateman 1999), while other proteins help with the formation of the triple helix itself (LH1,LH3 and the LH2-FKBP65 complex) (Eyre and Weis 2013; Lim et al. 2017) and others help with the stabilization of the triple helix (P3H1–CRTAP–CYPB complex and HSP47) (Duran et al. 2015; Eyre and Weis 2013; Lim et al. 2017).

Protein disulphide isomerase (PDI) catalyzes the formation of intrachain disulphide bonds in the non-collagenous C- and N-propeptide domains which helps to stabilize the procollagen trimers. Interchain disulphide bonds promote the association of the collagen chains at the C-terminus which is essential for their correct alignment and triple helix formation (Boudko et al. 2012; Koivu and Myllylä 1987). The importance of this step is shown by OI C-propeptide mutations where collagen is subjected to degradation (Lamandé et al. 1995). This is accompanied by the upregulation of binding immunoglobin protein (BiP) and glucose-regulated protein 94 (GRP94) molecular chaperones, which together with Calnexin, contribute to C-propeptide folding (Chessler and Byers 1993).

### Proline hydroxylation

PDI also serves as a subunit for the enzyme prolyl 4-hydroxylase (P4H), the latter of which hydroxylates triple helix prolines. This is essential for the formation of the procollagen triple helix and determines its stability which is evident by the correlation of its hydroxylation level with thermal stability (Burjanadze 1979). The conversion of peptide bonds in proline residues from cis to trans conformation takes place by peptidylproline cis–trans isomerase (PPIase), which further facilitates triple helix formation (Bächinger 1987).

In addition to 4-hydroxyproline, collagen also contains 3-hydroxyproline, the functional significance of which is much less understood. This is distinctly found in α1(Pro986) and α2(Pro707) collagen type I chains where hydroxylation takes place by *LEPRE1* encoding prolyl 3-hydroxylase 1 (P3H1) (Lim et al. 2017). P3H1 forms a heterotrimeric complex together with cartilage associated protein (CRTAP) and cyclophilin B (CYPB), encoded by *CRTAP* and *PPIB* respectivel*y*. This complex is shown as part of the posttranslational modification in Fig. 1a. Attention to the role of prolyl 3-hydroxylation in collagen integrity was initially triggered by the discovery of *CRTAP* mutations as cause for OI, which was demonstrated by collagen overmodification (Morello et al. 2006). The same effect was subsequently reported as a consequence of mutations in *LEPRE1* and *PPIB*, clearly pointing to a delay in collagen folding (Cabral et al. 2007; van Dijk et al. 2009a, b). However, upon defects in proteins of this complex, residual amount of 3-hydroxyproline can still often be observed (Marini et al. 2010). This can depend on the effect of the genetic defect, as shown by the presence of 85% 3-hydroxyproline in a patient in which the ER retention sequence (KDEL) was absent in LEPRE1 (Takagi et al. 2012). Regarding CYPB, it has been reported that mutations in *PPIB*, leading to no protein production, lead to a more severe OI phenotype compared to mutations that result in protein alterations; it is unclear if this relates to the level of residual 3-hydroxyproline (Rush et al. 2014). Interestingly, in this complex CRTAP and P3H1 stabilize each other and are thus both needed to form a stable protein complex, while CYPB appears to be independent of both (Pyott et al. 2011; van Dijk et al. 2009a, b). Concerning the mechanism of the complex, different functions have been attributed. The crystal structure of synthetic collagen revealed that the 3-hydroxyl group points out from the triple helix suggesting that it may serve as a recognition point for other proteins which may be involved in collagen fibrillogenesis (Schumacher et al. 2006). In this complex, CYPB has been found to have PPIase activity but it is unclear to which extent this contributes to the collagen abnormalities in relation to its function in the prolyl-3 hydroxylation complex (Homan et al. 2014; Ishikawa et al. 2009).

### Lysine hydroxylation

The hydroxylation of lysine residues in procollagen is also a necessary condition for the production of a functional molecule. This is performed by the *PLOD1-3* genes encoding lysyl hydroxylase 1–3 (LH1–3) which convert lysine residues to hydroxylysine (Hyl). Lysyl oxidases subsequently use the Hyl residues to generate pyridinoline cross-links which affect the properties of the bone tissue. Each LH functions in a specific location in procollagen. For instance, LH1 and LH3 hydroxylate lysine residues in the triple helix, while LH2 forms Hyl residues in the telopeptide regions, allowing intermolecular cross-links to take place between collagen fibrils (Gjaltema et al. 2016; Leal et al. 2018; Takaluoma et al. 2007). In recent studies, it has been postulated that LH2 forms a complex with FKBP65 which regulates its hydroxylase activity. The LH2-FKBP65 complex together with HSP47 is shown in Fig. 1a. FKBP65, FK-506-binding protein encoded by *FKBP10*, is a peptidyl prolyl isomerase and also a type I procollagen chaperone (Barnes et al. 2012; Lim et al. 2017). Gjaltema et al*.* showed that FKBP65 binds selectively to LH2 to promote the dimerization of LH2, and therefore, its function (Gjaltema et al. 2016). Moreover, Duran et al*.* identified the heat shock protein 47 (HSP47) and BiP as additional components of the FKBP65-LH2 complex (Duran et al. 2017).

In specific, HSP47 was found to inhibit LH2 access to the hydroxylation site, while FKBP65 promoted dimerization of LH2 and thus an increase in hydroxylation of type I procollagen (Duran et al. 2017). Proper lysine hydroxylation is essential for the activity of lysyl oxidase (LOX), an amine oxidase which deaminates specific lysine and hydroxylysine residues to form allysines in the telopeptide domains (Kagan and Li 2003). This subsequently facilitates the formation of collagen fibrils in the ECM as later discussed.

Considering the joint action of LH2 and FKBP65, it is not surprising to find similarities in the phenotypic presentation of patients with pathogenic mutations in *PLOD2* and *FKBP10,* respectively. As expected, mutations in *PLOD2* lead to underhydroxylation of the telopeptide lysines in bone type I collagen (Schwarze et al. 2013). Patients with these mutations are classified as having Bruck type 2 syndrome. Bruck syndrome is a rare autosomal recessive disease with great clinical overlap with OI, with the exception of congenital joint contractures and pterygia (Gjaltema et al. 2016; Kelley et al. 2011). Mutations in *FKBP10* on the other hand, lead to both Bruck syndrome type 1 and recessive, moderately severe OI type III. The *FKBP10* mutations probably lead to these diseases due to the resulting inability of LH2 dimer formation and the ensuing underhydroxylation of the procollagen type I telopeptides, which leads to its slight delayed secretion (Gjaltema et al. 2016; Schwarze et al. 2013). However, no specific location or type of mutation in this gene has been identified as specifically causative for the two diseases, and similarly to OI, phenotypic variability is seen even within families (Schwarze et al. 2013; Setijowati et al. 2012). Notably, mutations in *PLOD1* lead to a different connective tissue disease, the kyphoskoliotic type of Ehlers Danlos syndrome (EDS) (type 8) (Yeowell and Steinmann 1993).

### Chaperone activity of HSP47 and FKBP65

Collagen processing is also mediated by the two chaperone proteins HSP47 and FKBP65 which interact with each other and LH2 (Duran et al. 2017). HSP47 is produced by *SERPINH1*, it is induced by heat and is co-expressed with collagen (Ishida and Nagata 2011). HSP47 binds to both unfolded propeptides and the folded triple helix but has been described to preferentially bind the latter (Koide et al. 1999). Being an ER-resident protein, it binds to procollagen during its import in the ER in a pH-dependent manner (Satoh et al. 1996). While the pH is neutral in the ER, it is lower in the Golgi where the procollagen is transported, which allows its dissociation from the HSP47 (Nakai et al. 1992). HSP47 is transcriptionally induced by transforming growth factor β1 (TGFβ1) and its protein expression has been reported to be upregulated in response to collagen mutations (Yamamura et al. 1998). Additionally, *SERPINH1* mutations not only decrease levels of HSP47, but they also reduce the protein levels of FKBP65. *FKBP10* mutations do not reciprocate this effect on HSP47 (Duran et al. 2015).

Patients with absent HSP47 still produce normal, although less stable, type I collagen, but with slightly delayed secretion (Christiansen et al. 2010). The same effect is reported for *FKBP10* mutations (Barnes et al. 2012). This suggests that both FKBP65 and HSP47 probably affect collagen biosynthesis after the posttranslational modification stage performed among others by the prolyl-3 hydroxylation complex (Duran et al. 2015). However, a recent study presented an OI case with a homozygous *SERPINH1* substitution in which collagen type I showed overmodification despite normal folding and secretion. This was combined with higher expression level of several procollagen chaperones. It was shown that the mutant HSP47 protein had less affinity for collagen type I, which may possibly lead to local unwinding of the triple helix by lack of triple helix stability. In this way procollagen may be still subjected to overmodification (Syx et al. 2021).

### Calcium homeostasis-dependent chaperone activity

The process of biosynthesis of type I collagen depends heavily on intracellular calcium; Ca^2+^ binding to the C-terminal globular domain of procollagen stabilizes the process of collagen trimerization and folding by assisting the formation of interchain hydrogen and disulfide bonds. In this process, several chaperone proteins are regulated by the Ca^2+^ fluctuations in the ER including PDI, CYPB, LH1, LH2 and FKBP65. One of the channels affecting the ER Ca^2+^ flow is the TRIC-B, shown in Fig. 1a, an intracellular monovalent cation channel, encoded by *TMEM38B*. A disruption of the ER Ca^2+^ homeostasis by *TMEM38B* mutations potentially influences collagen biosynthesis by affecting collagen chaperone proteins (Cabral et al. 2016). Patient cells show decreased collagen secretion due to retention of the defective collagen, which is reported to be less stable owing to decreased helical lysine hydroxylation but increased telopeptide hydroxylation even though increased LH1 and decreased FKBP65 expression was noted respectively (Cabral et al. 2016). Together with TRIC-A and TRIC-B, Ca^2+^ release is regulated by the inositol triphosphate receptors (IP_3_R) and ryanodine receptors (RyR). The sarcoplasmic-ER Ca^2+^–ATPase type 2b (SERCA2b) plays a role in calcium uptake. SERCA2b activity requires its coupling to the ER membrane translocation associated membrane protein 2, TRAM2, for collagen translation (Stefanovic et al. 2004). The two TRIC proteins are needed to maintain the Ca^2+^ homeostasis by providing counter–ion currents to facilitate the Ca^2+^ release from the intracellular stores by iP_3_R and RyR (Wang et al. 2019). No differences in the expression levels of IP_3_R and SERCA2b are noted in OI patients with *TMEM38B* mutations (Cabral et al. 2016).

## Anterograde vesicle transport

After the posttranslational modifications and the triple helix folding have been completed in the ER lumen, procollagen is transported from the ER to the Golgi apparatus via vesicle transport (Stephens 2012). However, the transport of the procollagen triple helix is different than most other proteins. This is due to the large size of the triple helix, which is estimated to reach 400 nm. Effective transport may be further complicated by the rigid rod-like element of collagen. This poses a problem since vesicles typically have a diameter of only up to 90 nm (Raote et al. 2018). For this reason, it has been hypothesized that procollagen secretion takes place in large coat protein II (COPII) vesicles with a diameter between 400 and 1200 nm (Mccaughey et al. 2019).

Anterograde transport, the trafficking of proteins from the ER to the Golgi, is accomplished with the COPII vesicles which are formed by the concerted action of different proteins, shown in Fig. 1b. The process starts at the ER exit sites where the ER resident SEC12 protein stimulates the exchange of GDP with GTP in the small GTPase SAR1, as a result of which, the SAR1-GTP protein is imbedded in the outer lipid bilayer of the ER. It then recruits the SEC23/SEC24 heterodimer by interacting with SEC23, forming the inner coat of the COPII vesicle. In the last step, SEC13/SEC31 binds SEC23/SEC24 heterotetramers in order to activate SEC23, whose GAP activity leads to the hydrolysis of the SAR1-bound GTP; in this structure SEC13/SEC31 form the outer COPII coat and they are crucial for the induction of curvature needed for the spherical vesicle shape (Malhotra and Erlmann 2015).

Cargo proteins in COPII vesicles are commonly recruited by SEC24 by binding to its specific cargo-binding site. However, this is not the case with soluble proteins which need receptor proteins to anchor them to the vesicle coat (Gomez-Navarro and Miller 2016). In the case of the bulky procollagen, packaging to vesicles requires the transmembrane proteins transport and Golgi organization 1 (TANGO1) and cutaneous T cell lymphoma-associated antigen 5 (cTAGE5) proteins. TANGO1 possesses a cytoplasmic, transmembrane and ER luminal domain; the cytoplasmic part interacts with SEC23 and cTAGE5 whereas the luminal domain binds procollagen through HSP47, as shown in Fig. 1b (Ishikawa et al. 2016; Widmer et al. 2012). Although this interaction has been extensively studied in collagen type VII, it has been found that loss of TANGO1 leads to procollagen type I retention in the ER, which clearly extends its involvement to multiple collagen types in agreement with its role in large protein transport (Ma and Goldberg 2016; Ríos-Barrera et al. 2017; Saito et al. 2011). According to Raote et al*.* TANGO1 forms a ring around the ER secretion point which prevents the recruitment of the outer COPII coat, which ensures that the whole procollagen triple helix is encapsulated before the vesicle closes (Raote et al. 2018). TANGO1 has been also proposed to interact with SEC16 at the ER exit site which influences the GAP activity of SEC23 (Kung et al. 2012); this can also regulate vesicle closure when an appropriate size has been reached (Maeda et al. 2017). In this way TANGO1 acts as cargo receptor for collagen and facilitates the assembly of mega COPII vesicles (Raote et al. 2018; Yuan et al. 2018). Interestingly, another factor that may promote this process, is the ubiquitination of some SEC31 molecules by the ubiquitin ligase Cullin3 and its adaptor protein KLHL12 (Jin et al. 2012). Even though ubiquitination is primarily known as a protein degradation mechanism, it can also affect protein function. Although the mechanistic details remain to be elucidated, the overexpression of Cullin3–KLHL12 has shown to produce COPII structures of a sufficient size to accommodate collagen (Jin et al. 2012). The concept of collagen transport by vesicles is challenged by findings of a recent study by McCaughey et al*.* which proposes the possibility of a direct connection between the ER and Golgi apparatus for the transfer of procollagen. This connection is suggested to involve the budding formation of COPII vesicles at the ER site in close proximity to the Golgi apparatus, where TANGO1 mediates their fusion to the ER–Golgi intermediate compartment. After scission, the formed compartment takes on the characteristics of the Golgi apparatus (Mccaughey et al. 2019).

The significance of the anterograde transport in skeletal diseases is exemplified by multiple diseases with genetic defects in genes encoding for components of the COPII procollagen transport (Ohisa et al. 2010). In mammals, gene duplications during evolution have created several paralogs of the COPII components, mutations in which have been found to lead to different syndromes. Loss of functional SEC23A leads to cranio-lenticulo-satural dysplasia (CLSD), characterized by skeletal defects, facial dysmorphisms hypertelorism and Y-shaped cataracts (Boyadjiev et al. 2011). Mutations in *SEC23B* lead to congenital dyserythropoietic anemia type II (CDA-II). The disorder shows ineffective erythropoiesis, abnormal bone marrow morphology and anomalous erythrocyte membrane proteins (Ohisa et al. 2010; Schwarz et al. 2009). *SEC23B* mutations also lead to Cowden syndrome which presents increased predisposition to different cancer types and macrocephaly (Yehia et al. 2015). Pathogenic mutations in another inner COPII coat protein encoding gene, *SEC24D*, lead to a syndromic form of OI with a disease presentation between CLSD and Cole-carpenter syndrome. Patients show bone deformities, multiple fractures, craniofacial malformations, short stature, but no sign of cataracts (Garbes et al. 2015). Mutations in *SAR1B* cause chylomicron retention disease (CMRD), characterized by enterocyte failure to secrete large lipoprotein particles. Patients show slow growth and weight gain, frequent diarrhea, steatorrhea and hypocholesterolemia (Ohisa et al. 2010). Regarding the outer coat components, mutations in *SEC31A* cause a neurodevelopmental disorder with spastic quadriplegia, optic atrophy, seizures, and structural brain anomalies; this is accompanied by delayed growth, contractures and clubfoot. Finally, mutations in *TANGO1,* producing truncated or reduced levels of the protein, lead to a novel autosomal recessive syndrome. Dentinogenesis imperfecta, growth retardation, short stature, hearing loss, insulin-dependent diabetes mellitus and mild intellectual disability are reported (Lekszas et al. 2020). Recently, TANGO1 deficiency was reported to lead to embryonic lethality with skeletal dysplasia and striking undermineralization (Guillemyn et al. 2021). Clearly, impaired COPII transport leads to diverse skeletal abnormalities in combination with many different phenotypic features. It is tempting to assume that this is related to the distinct profile of vesicle cargo proteins affected by each genetic abnormality, including procollagen type I.

## Retrograde vesicle transport

Newly synthesized proteins are transported from the ER to the Golgi apparatus where they can be sorted to their destination (Aoki et al. 2009). However, some of these proteins have to be transported back to the ER where they exert their function; this happens by retrograde transport. Unlike COPII-coated vesicles in anterograde transport, retrograde transport of proteins from the Golgi to the ER takes place in vesicles with a COPI coating. In contrast to the separate inner and outer coat structure found in COPII vesicles, COPI coating is composed of seven subunits (α-COP, β’-COP, ε-COP, β-COP, δ-COP, γ-COP and ζ-COP), shown in Fig. 1c (Arakel and Schwappach 2018). The assembly of the COPI-coated vesicles at the Golgi membrane starts with the activation and binding of the small GTPase ARF1 to the membrane; in mammalian cells the protein GBF1 has been suggested to catalyze the association of GTP with ARF1 (Kawamoto et al. 2002). This leads to the assembly of the coat complex in which three haptameers make up its basic unit. Vesicle budding is allowed when the GTP in ARF1 is hydrolyzed back to GDP. Sorting of proteins back to the ER is based on the presence of the common retrograde KKXX motif, to which the α-COP and β’-COP subunits are described to bind (Letourneur et al. 1994; Raykhel et al. 2007). In addition, certain proteins such as the KDEL receptor, also mediate the sorting of ER proteins by binding COPI and functioning as an adaptor as shown in Fig. 1c (Shibuya et al. 2015). Receptors of this family include KDELR1, KDELR2 and KDELR3, each consisting of 7 transmembrane domains. These receptors recognize proteins that have to be returned back to the ER by binding to their C-terminal Lys–Asp–Glu–Leu (KDEL) motif (Capitani and Sallese 2009); variations of the KDEL sequence also exist. The binding of the receptor to the KDEL(-like)-containing protein is pH-regulated. The acidic lumen of the Golgi promotes the interaction of the KDEL receptor with the KDEL motif in the cargo protein. This contributes to the formation of the COPI vesicles and the subsequent retrograde transport. Once found in the ER, the less acidic environment leads to the release of the protein cargo from the KDEL receptor, the latter of which is then recycled back to the Golgi (Wu et al. 2020).

Considering that collagen type I is heavily subjected to many types of posttranslational modifications, it is safe to conclude that correct collagen maturation requires substantial contribution from many chaperones, many of which are proteins that reside in the ER. For this reason, it is not surprising that the retrograde protein transport, which facilitates chaperone regulation, is emerging as a key mechanism in OI pathology. We recently showed that mutations in KDELR2 lead to OI type II and III, which was accompanied by a decrease of procollagen chaperone proteins HSP47 and FKBP65 in fibroblasts of the patients. This also correlated with decreased secretion of procollagen type I and the lack of proper collagen fiber assembly. Notably, HSP47 was found associated with the extracellular monomeric and multimeric collagen molecules (van Dijk et al. 2020). HSP47 and procollagen are known to dissociate from each other at ER exit sites before collagen’s anterograde transport; it remains to be investigated how this process is disrupted in cells with KDELR2 mutations (Omari et al. 2020).

Both HSP47 and FKBP65 have a KDEL-like domain (Table 2) which can account for their decreased expression in patients with *KDELR2* mutations. However, given that the expression of FKBP65 has been reported to be influenced by the availability of HSP47, it is not clear to which extent this is the case in these patients (Duran et al. 2015). In addition to HSP47 and FKBP65, there are several more proteins with a KDEL(-like) motif which play a role in collagen regulation (Table 2). Of these, LEPRE1 and P4HB have a KDEL motif and mutations in both are known to cause OI and Cole–Carpenter syndrome respectively. Other collagen chaperones with a KDEL(-like) motif have not been identified to cause OI/bone-related disorders.

**Table 2:** KDEL motif-containing proteins implicated in collagen biosynthesis

Gene	Motif	Protein	Disease association
*SERPINH1*	RDEL	HSP47	OI
*GRP78*	KDEL	HSPA5 / BIP	No specific associated disease
*P3H2*	KDEL	LEPREL1	Cataract
*P3H3*	REEL	LEPREL2	No specific associated disease
*P4HB/PDIA1*	KDEL	PDIA1	Cole-Carpenter syndrome
*FKBP10*	HEEL	FKBP65	Bruck syndrome
*P3H1*	KDEL	LEPRE1	OI
*HSP90B1*	KDEL	GRP94	No specific associated disease
*PDIA2/PDIP*	KEEL	PDIA2	No specific associated disease
*PDIA4*	KEEL	PDIA4	No specific associated disease
*PDIA5*	KEEL	PDIA5	No specific associated disease
*PDIA6*	KDEL	PDIA6	No specific associated disease

*PDI* protein disulphide isomerase

Another protein involved in retrograde transport is the neuroblastoma-amplified gene protein, NBAS (Aoki et al. 2009). NBAS is a peripheral protein, which forms a subunit of the Syntaxin 18 (STX18) complex by binding to the p31 part of STX18 as shown in Fig. 1c. Syntaxin 18 plays a role in the membrane fusion of retrograde transport vesicles with the acceptor ER compartment (Balasubramanian et al. 2017; Iinuma et al. 2009; Segarra et al. 2015). NBAS has also been presented to play a role in nonsense mediated mRNA decay (NMD) which selectively degrades mRNA with a premature stop termination codon to prevent accumulation of truncated proteins that could interfere with cellular functions (Balasubramanian et al. 2017). NMD also has a role in regulating endogenous mRNA expression of genes with an osteogenic function (Longman et al. 2013). NBAS has been associated with an autosomal recessive disorder called short stature with optic atrophy and Pelger–Huët anomaly (SOPH) syndrome. Patients have features such as short stature, acute liver failure, optic nerve atrophy, facial dysmorphisms and Pelger–Huët anomaly of leucocytes (Balasubramanian et al. 2017; Maksimova et al. 2010). Balasubramanian et al*.* presented two patients having heterozygous mutations in *NBAS* showing OI characteristics such as short stature, bone fragility and developmental delay (Balasubramanian et al. 2017). In addition, the patients also presented atypical OI features such as optic atrophy, immunodeficiency and abnormal liver function tests, which overlap with the SOPH syndrome phenotype. Fibroblasts from a patient showed decreased collagen secretion and variable shaped collagen fibrils. It is plausible that NBAS defects lead to bone abnormalities by disturbed retrograde transport and/or compromised NMD targeting of bone-related genes. Recently, mutations in *ARCN1*, encoding the coatomer subunit delta of COPI, have also been shown to lead to a syndrome of craniofacial deformities which correlated with ER stress and reduced collagen secretion in *ARCN1* knockdown fibroblasts (Izumi et al. 2016). Regarding other bone-related diseases caused by defects in the components of the COPI vesicles, mutations in the *COPA* gene cause COPA syndrome, an autosomal dominant autoimmune disease, characterized by arthritis and inflammation of the lungs, kidneys and joints (Patwardhan and Spencer 2019).

## ER stress response in OI

In addition to feeding protein trafficking to the Golgi, ER is a main cell site for protein quality control which is consistent with the high abundance of ER-resident chaperones. Exposure of hydrophobic regions, cysteine residues and protein aggregates signal folding errors to chaperones such as BiP, Calnexin, Calreticulin, GRP94 and PDI which bind to the misfolded proteins to retain them in the ER (Ellgaard and Helenius 2003); the latter 2 are KDEL(-like) motif proteins. Accumulation of misfolded proteins can be sensed by the ER, in response to which the unfolded protein response (UPR) is activated. UPR ensures that the abnormal protein stays in the ER until its conformation can be improved by the chaperones. If the protein is defective beyond repair, it is destined for degradation. This can happen by ER-associated degradation (ERAD), by which the protein is transported to the cytoplasm where it is ubiquitinated and subsequently degraded by the proteasome. An alternative pathway is autophagy by which the irreversibly damaged protein become degraded in lysosomes.

However, if the ER stress is prolonged, at a certain stage cell death can ensue (Tsai and Weissman 2010). The UPR mechanism stems from three different receptors on the ER membrane, activating transcription factor 6 (ATF6), inositol-requiring protein 1 (IRE1) and PKR-like ER kinase (PERK). Notably, the KDEL-containing protein BiP occupies the lumenal domain of all three receptors; increase in ER-unfolded proteins frees the receptors from BiP as the latter migrates to bind to the problematic proteins (Gardner et al. 2013). Activation of ATF6 by ER stress results in its translocation to the Golgi where it is sequentially cleaved by the site metalloprotease 1 (S1P) and 2 (S2P), which gives rise to the active transcription factor which can then regulate the expression of UPR-related genes, such as the cytoplasmic X-box binding protein 1 (XBP1), encoded by the corresponding gene *XBP1*, and CCAAT/enhancer-binding protein homologous protein (CHOP), encoded by the gene *DDIT3* (DNA damage Inducible Transcript 3) (Yang et al. 2020). IRE1 activation leads to the removal of an intron from XBP1 mRNA, producing the isoform XBP1s; XBP1s regulates chaperone and ERAD protein expression (Chen and Brandizzi 2013). The activation of PERK also has an important role as it phosphorylates eIF2α which can decrease the ER load during ER stress as it inhibits protein translation. However, the translation of activating transcription factor 4 (ATF4) is allowed to take place in agreement with its role in gene expression regulation mediating cell death (Wortel et al. 2017).

Given that severe OI is largely a disease of misfolded protein, it can be easily deduced that ER stress is part of the underlying mechanism. Even though OI was originally solely considered as a disease of structural defects, it has been long ago recognized that the activation of ER stress and of related pathways also deserve attention in OI development. This is evident by the identification of OI causative genes in components participating in the UPR pathway. One of these is the site 2 metalloprotease (S2P), encoded by the membrane-bound transcription factor peptidase site 2 (*MBTPS2*). This is a cleaving protease located on the Golgi membrane, involved in the ER stress response and cholesterol metabolism (Lindert et al. 2016). One of the proteins cleaved by S2P upon ER stress is the old astrocyte specifically induced substance (OASIS), encoded by the cAMP responsive element binding protein 3-like 1 (*CREB3L1*), also a rare cause of OI, shown in Fig. 1d. After cleavage, this transcription factor is shuttled to the nucleus to activate the transcription of genes involved in the ER stress response pathway or UPR pathway. The cleaved OASIS also binds to the *COL1A1* promoter via SMAD4 to increase collagen type I expression, shown in Fig. 1d (Lindert et al. 2016). Keller et al*.* also presented OASIS as playing a possible upstream role in the formation of the COPII complex, through regulation of the COPII inner coat component SEC24D (Keller et al. 2018*).*

Mutations in *MBTPS2* lead to X-linked moderate OI as well as other syndromes depending on the location of the mutation (Lindert et al. 2016). These include IFAP/BRESHECK, characterized by ichithyosis follicularis, atrichia and photophobia (Corujeira et al. 2013), keratosis follicularis spinulosa decalvans (KFSD), characterized by scarring alopecia of the scalp, eyebrows and axillae (Malvankar and Sacchidanand 2015), and Olmsted syndrome, characterized by bilateral mutilating transgradient palmoplantar keratoderma and periorificial keratotic plaques (Duchatelet and Hovnanian 2015). Of these only the first shows skeletal abnormalities and vertebral anomalies, including scoliosis and chest hypoplasia. In X-linked OI, reduction of OASIS cleavage fragments was observed, which subsequently led to less type I procollagen secretion. In addition, the expression of both *CREB3L1* and its nucleus complex partner *SMAD4* were reduced during osteoblast differentiation (Lindert et al. 2016). The severity of the OI phenotype can differ enormously depending on the location of the mutations and their allelic status; in the first reported families, biallelic mutations lead to embryonic lethality whereas in the latter two, biallelic patients were reported to survive infancy despite severe skeletal deformities (Cayami et al. 2019; Guillemyn et al. 2019; Symoens et al. 2013). Interestingly, members of the described family with monoallelic mutations in *CREB3L1* presented a mild OI clinical picture with blue sclera, osteopenia and few fractures (Keller et al. 2018). It is suspected that less functional OASIS leads to less expression of SEC24D which in turn disrupts COPII formation and subsequently gives way to less efficient secretion of type I collagen and other bone matrix proteins in osteoblasts (Keller et al. 2018).

Activation of UPR has been also demonstrated in collagen type I mutations. BiP was originally reported to increase and specifically associate with C-terminal procollagen mutations, and not in fibroblasts with mutations in the helical domain (Chessler and Byers 1993; Lamandé et al. 1995). However, a more recent study showed that BiP, as well as phosphorylated PERK and ATF4 expression and splicing of XBP1, can be increased in fibroblasts with helical mutations. In the same study, the defective collagen was shown to accumulate in the cell ER and lead to autophagy and apoptosis. Treatment with the FDA-approved chemical chaperone 4-phenylbutyrate, a histone deacetylase inhibitor, could reduce UPR stress, and enhanced protein clearance by stimulating autophagy (Besio et al. 2018). Enlarged ER due to the retention of misfolded collagen has been also shown in OI patient fibroblasts with mutations in *CRTAP*, *PPIB*, and *P3H1* impairing prolyl-3 hydroxylation. This generally coincided with activation of UPR, as shown by the upregulation of BiP, phosphorylated PERK and ATF4, as well as autophagy and apoptosis. Also, here, 4-phenylbutyrate decreased the size of ER cisternae and was able to reduce UPR and apoptosis (Besio et al. 2019). This highlights abnormal collagen trafficking as a common therapeutic avenue for different genetic causes of OI which can be counteracted by alleviating ER stress.

Given the promising results of the chemical chaperones in alleviating OI ER stress, it is important to comprehend the underlying mechanism which can lead to more targeted interventions. Currently, little is known about how the different ER stress branches directly affect the different steps of collagen type I regulation. However, a recent study, from DiChiara et al*.*, interestingly showed that forced expression of *XBP1* in OI fibroblasts with a glycine substitution, markedly increased *COL1A1* expression and that this even improved mutant collagen type I folding. On the contrary, activation of the ATF6 pathway had no effect on collagen secretion. It will be exciting to see if these findings can be reproduced in OI osteoblasts and other dominant negative collagen mutations. In summary, OI mutations affecting collagen type I structure can have different effects. Detection of overmodified collagen shows that it is possible for collagen to escape the quality control of the cells (Van Dijk et al. 2009a, b). In other cases, this leads to chronic ER stress and reduced production of collagen (Lindert et al. 2018; van Dijk et al. 2020). The trafficking of unfolded COL1α2 to the Golgi for subsequent lysosome-dependent degradation has been also reported in the absence of COL1α1 chains (Gotkin et al. 2004). Future investigations may be able to determine how this outcome depends on specific collagen defects.

## Extracellular processing

Procollagen trafficking after the Golgi complex entails its destination to the extracellular matrix. At the Golgi apparatus, procollagen type I is still in possession of a propeptide at each of its two ends which prevents its premature assembly into fibrils (Mienaltowski and Birk 2014). Once secreted to the ECM, procollagen type I is exposed to the proteolytic activity of BMP1/tolloid-like proteinases and ADAMTS 2,3 and 14 which cleave off the C- and N-procollagen domains respectively. After cleavage, the triple helix remains in the center flanked by short non-collagenous sequences called telopeptides. The fully formed type I collagen molecules then assemble spontaneously into quarter-staggered arrays which allows their close packing in the collagen fibrils (Gelse et al. 2003; Mienaltowski and Birk 2014). This process is shown in Fig. 1e.

Despite the fact that extracellular processing takes place in the ECM, it is still dependent on proper intracellular trafficking which ensures that procollagen is correctly primed for ECM processing. This means that the hydroxylation of lysine residues in the procollagen triple helical and telopeptide regions in the ER by LH enzymes is paramount for the integrity of the collagen fibrils since they serve as recognition points for LOX. This allows the formation of cross-links between telopeptides and the triple helix of collagen molecules, which promotes the arrangement of the mature collagen molecules into fibrils with a distinctive 67 nm axial periodicity (Tzaphlidou 2001). The detrimental consequences of lack of LOX activity on collagen fibrillogenesis are seen after incubation of osteoblasts with the enzymatic inhibitor, β-aminopropionitrile (BAPN), as a result of which crosslinks and osteoblast differentiation are dysregulated (Turecek et al. 2008). This is also in agreement with the defective crosslinking seen in patients with OI/Bruck syndrome patients with *PLOD2* and *FKBP10* mutations (Gistelinck et al. 2020). This also highlights the need for impeccable coordination with COPI trafficking which delivers the FKBP65 back to the ER to make it available for procollagen hydroxylation. Interestingly, loss of function mutations in *LOX*, are not involved in OI, but in thoracic aortic aneurysms and dissections (Corradi et al. 2015).

Certain connective tissue disorders occur by mutations in enzymes regulating procollagen cleavage. Recessive mutations in *ADAMTS2* lead to dermatosparaxis EDS type 7, characterized by extreme skin fragility in combination with short limbs, hand and feet, craniofacial features and osteopenia while recessive mutations in *ADAMTS3* lead to Hennekam lymphangiectasia–lymphedema syndrome 3, characterized by primary lymphedema in the lower extremities (Malfait et al. 2017; Mead and Apte 2018). Mutations in *BMP1* cause autosomal recessive OI due to defects in the extracellular processing of procollagen type I, more specific defective C-propeptide removal and potential subsequent cross-linking defects (Eyre and Weis 2013; Marini et al. 2014). Patients have fragile bones, but a high mineral density, recurrent fractures, generalized bone deformity and osteopenia (Eyre and Weis 2013; Pollitt et al. 2016). Similarly, OI patients with C-propeptide cleavage site mutations are also reported to have high bone mass (Lindahl et al. 2011). Interestingly, BMP1 also processes LOX, which makes it indispensable in ECM collagen processing (Uzel et al. 2001).

Although it is established that the transport of procollagen to the plasma membrane is mediated by microtubule-associated secretory vacuoles budding off from the Golgi, it is unclear how collagens find their way to their allocated location and at which point their sorting route diverges (Leblond 1989; Weinstock and Leblond 1974). For example, while procollagen I is targeted to all ECM sites of the cell, procollagens IV and VII are destined to the cell basal membrane (Malhotra and Erlmann 2015). TANGO is known to recognize collagen types I, IV and VII, although perhaps not with the same efficiency (Malhotra and Erlmann 2015). Future studies will show at which stage of collagen trafficking, the distinct localization of different collagen types is determined. It has been suggested that the formation of new collagen fibers occurs in fibripositors which are extensions of the plasma membrane (Canty et al. 2004; Kadler et al. 2008). It is important to mention that the formation of fibrils does not only depend on the intrinsic collagen properties but also on other molecules which bind to collagen; small leucine-rich repeat proteoglycans (SLRPs) such as decorin, biglycan, fibromodulin and lumican can affect collagen fibril diameter (Chen and Birk 2013). Fibronectin and integrins are also described as major regulators of fibril assembly as shown by the disruption of fibrillogenesis when their binding to collagen is blocked (Li et al. 2003; McDonald et al. 1982). Fibril assembly determines the mechanical properties of bone tissue not only by the quality of the collagen fibers themselves but also by mineralization (Nair et al. 2013; Wenger et al. 2007).

## Discussion

The identification of numerous genetic causes of OI in the last years has not only improved patient diagnosis, but in parallel has also given insight in multiple levels of collagen regulation and the clinical consequences arising from their dysregulation. It has been suggested that a mechanism-based classification of OI would provide a basis for extension of the existing classification system, which could also serve in the future as a basis for the development of treatment (Forlino and Marini 2016). This review recapitulates our understanding of collagen trafficking, highlighting the significance of anterograde and retrograde transport. Based on the current state of knowledge in this review of the literature (Table 1), limited conclusions can be drawn on genotype–phenotype correlations which points to the fact that we still need to understand critical factors in the regulating mechanism as well as intrafamilial and interfamilial OI variation (Forlino and Marini 2016; Zhytnik et al. 2020). This variation is also reflected in the international Osteogenesis Imperfecta Variant Database where individual mutations can lead to different clinical OI types (https://oi.gene.le.ac.uk). A general trend supports an occurrence of a mild phenotype arising from less collagen production (null-allele) and a more severe phenotype as a result of structural collagen abnormalities from collagen defects or defects in collagen-modifying enzymes (Zhytnik et al. 2019). Also, in certain cases, the severity of the phenotype can be explained by the effect of the mutation. For example, OI patients with *BMP1* mutations with residual activity of the BMP1 protein have a milder clinical presentation compared to patients with *BMP1* null mutations (Pollitt et al. 2016). However, the effect of the mutation still does not correlate with high bone mineral density which is found in some of the patients. Owing to the lack of clear genotype–phenotype correlation, the testing of all known OI genes is generally recommended in new OI patients.

Even though the clinical variability is a well-recognized OI feature which has been attributed to modifier genes, almost no study exist addressing this issue (Fitzgerald et al. 2013). The use of Genomic Mismatch Scanning-based method that physically identifies shared genomic regions between family members, revealed loci in Amish families descended from a common founder, that can potentially serve as modifier genes in OI. One of the loci was *PTGS2* encoding cyclooxynase 2 (COX2), which is involved in bone development by expression in osteoblasts (Brooks et al. 2009). Regarding clinical variation in OI caused by dominant negative collagen mutations, it can be hypothesized that different levels of chaperone expression may be able to regulate structural abnormalities which may consequently affect phenotypic presentation. However, experimental evidence supporting influence of chaperone levels on collagen overmodification or phenotypic presentation of dominant negative collagen OI is still lacking (Besio et al. 2018). The presence of miRNAs regulating osteogenesis may also influence OI progression which warrants investigation (NCT04009733; clinicaltrials.gov) (Kaneto et al. 2014). Considering the complexity of the OI mechanism, we recommend the use of the Sillence clinical classification with a note of the genetic defect as the most effective way to communicate the disease based on the current state of knowledge. We also hope that in the coming years the scientific community will deliver advancements towards explaining the clinical variability of OI.

In this review, only genes with a direct relation in the regulation of collagen were discussed. For this reason, the review did not address the OI causative genes *SERPINF1*, *FAM46A*, *SPARC, SP7*, *CCDC134*, *IFITM5*, *MESD*, *WNT1* and *PLS3*, although this does not exclude the possibility that they might influence collagen regulation. Absence of evidence for collagen-related regulation may be also attributed to the fact that certain genes may not have been sufficiently investigated in human bone tissue, which is the most relevant tissue in studying skeletal pathology in OI. This review is also preferentially focused on studies with human cells and diseases, so the insights of animal models in collagen trafficking were not discussed.

The trafficking of collagen is an intricate process and its smooth orchestration is essential for the composition and mechanical properties of the bone tissue (Canty and Kadler 2005). The formation of collagen fibrils is the end product of the transition of collagen in several cell compartments through which it is subjected in a controllable manner to gradual maturation. Recent findings on the anterograde and retrograde transport, stress the fact that correct collagen regulation does not only require its own effective transportation across the protein transport system of the cells but also the proper trafficking of chaperone molecules on which procollagen relies for its conformational integrity. This means that mechanisms contributing to collagen chaperone regulation, such as their retrograde transport back to the ER, warrant investigation in OI. This is particularly evident from the recent identification of the KDELR2 receptor as a causative gene for OI, which specifically facilitates the Golgi-ER recycling of KDEL(-like) motif-containing collagen chaperones (van Dijk et al. 2020). Not all mutated components of the COPII and COPI vesicles affect skeletogenesis, which may partially reflect their mechanistic impact on collagen regulation. The ER has been long recognized as the main location of posttranslational modifications for procollagen. In cases of collagen retention, ER stress pathways are activated, a common effect in both collagen and non-collagen mutations (Besio et al. 2019; Chessler and Byers 1993; Lamandé et al. 1995; Tsai and Weissman 2010). Interestingly, calcium depletion has been shown to stimulate the cell secretion of ER resident which can be attenuated by KDEL receptors the expression of which is increased under ER stress (Trychta et al. 2018).

This provides an exciting thought about the way calcium fluctuations in *TMEM38B* OI mutations could influence KDEL-containing OI chaperones, considering that they are also known to cause ER stress (Cabral et al. 2016).

Therapy for OI is currently not administered on a genetic defect type basis. OI patients are most commonly treated with orthopedic procedures aiming to rectify skeletal deformity and bisphosphonates, which are anticatabolic drugs for osteoporosis (Etich et al. 2020a). Bisphosphanates are analogs of pyrophosphate with affinity for bone hydroxyapatite to which they bind to inhibit osteoclast activity (Rogers et al. 2020). Also, medications in OI clinical trials such as BPS804 (NCT01417091; clinicaltrials.gov) and Romosozumab (NCT04545554; clinicaltrials.gov) (neutralizing anti-sclerostin antibodies), Denosumab (NCT01799798; clinicaltrials.gov) (neutralizing anti-RANKL antibody) and a combination of teriparitide (parathyroid hormone analog) with zolendronic acid (NCT01679080; clinicaltrials.gov) were originally not designed specifically for OI-related fractures. Similarly to bisphosphonates, denosumab also has antiresorptive activity as it blocks the RANKL ligand that stimulates osteoclastogenesis (Baron et al. 2011). On the contrary, medications of sclerostin antibodies and teripartide are considered to have an osteoanabolic effect. They both stimulate osteoblast function and survival; blocking of sclerostin inhibits WNT signaling which normally hinders osteoblast differentiation (Lewiecki 2014). Teriparatide, an analog of human parathyroid hormone, also stimulates osteoblast function by binding to its receptor (Bodenner et al. 2007).

An exception to that is Fresolimumab (NCT03064074; clinicaltrials.gov) (neutralizing anti-TGF-β antibody) which specifically targets TGF-β signaling in OI with CRTAP and COL1A2 defects (Grafe et al. 2014). TGF-β signaling is known to stimulate osteoblast differentiation (Bonewald and Mundy 1990). Considering the promising results of chemical chaperones in alleviating ER stress, we anticipate that this will be a promising therapeutic avenue for diverse defects in chaperone transport leading to ER procollagen retention (Besio et al. 2018). However, none of the published studies have shown improvement of collagen overmodification which characterizes structural defects in collagen (Besio et al. 2018; Gioia et al. 2017; Takeyari et al. 2020). Chemical chaperones can have different molecular effects; thus, it remains to be discovered how they specifically decrease ER stress and collagen accumulation in OI cells (Wong and Shoulders 2019). In addition, we envision that it could be possible in the future to treat collagen defects based on their type. A pharmacological approach enhancing collagen production could be ideal for patients with quantitative collagen mutations, whereas gene therapy could allow targeting of mutations leading to structural collagen defects.

Based on the above, it is clear that collagen transport is a central process in OI pathogenesis, the many stages of which influence the assembly of collagen fibrils, dictating bone quality. Chaperone regulation through retrograde transport deserves attention as a critical point affecting collagen maturation and ultimately bone development. We hope that the insights in these pathways will stimulate research towards this direction to further unravel the molecular sequence leading to bone fragility in OI.

## Data Availability

Not applicable.
